# Twelve-month effectiveness and safety of bictegravir/emtricitabine/tenofovir alafenamide in people with HIV from the Canadian cohort of the observational BICSTaR study

**DOI:** 10.1097/MD.0000000000037785

**Published:** 2024-04-19

**Authors:** Alexander Wong, Jason Brunetta, Joss De Wet, Ken Logue, Hugues Loemba, Taban Saifi, Dylana Mumm, Andrea Marongiu, Rebecca Harrison, David Thorpe, Benoit Trottier

**Affiliations:** a University of Saskatchewan, Regina, SK, Canada; b Maple Leaf Medical Clinic, Toronto, ON, Canada; c Spectrum Health, Vancouver, BC, Canada; d St Clair Medical Associates, Toronto, ON, Canada; e ByWard FHT Clinic, Ottawa, ON, Canada; f Gilead Sciences Canada, Inc., Mississauga, ON, Canada; g Gilead Sciences Europe Ltd, Uxbridge, UK; h Clinique de Médecine Urbaine du Quartier Latin, Montreal, QC, Canada.

**Keywords:** antiretroviral therapy, B/F/TAF, bictegravir, Canada, HIV, real-world evidence

## Abstract

The BICSTaR (BICtegravir Single Tablet Regimen) study is investigating the effectiveness and safety of bictegravir/emtricitabine/tenofovir alafenamide (B/F/TAF) in people with human immunodeficiency virus (HIV) treated in routine clinical practice. BICSTaR is an ongoing, prospective, observational cohort study across 14 countries. Treatment-naïve (TN) and treatment-experienced (TE) people with HIV (≥18 years of age) are being followed for 24 months. We present an analysis of the primary endpoint (HIV-1 RNA < 50 copies/mL; missing-equals-excluded [M = E]) at month 12 in the BICSTaR Canada cohort, including secondary (CD4 count, CD4/CD8 ratio, safety/tolerability) and exploratory (persistence, treatment satisfaction) endpoints. In total, 201 participants were enrolled in the BICSTaR Canada cohort. The analysis population included 170 participants (TN, n = 10; TE, n = 160), with data collected between November 2018 and September 2020. Of the participants, 88% were male, 72% were White, and 90% had ≥ 1 comorbid condition(s). Median (quartile [Q]1–Q3) age was 50 (39–58) years and baseline CD4 count was 391.5 (109.0–581.0) cells/µL in TN participants and 586.0 (400.0–747.0) cells/µL in TE participants. After 12 months of B/F/TAF treatment, HIV-1 RNA was < 50 copies/mL in 100% (9/9) of TN-active participants and 97% (140/145) of TE-active participants (M = E analysis). Median (Q1–Q3) CD4 cell count increased by +195 (125–307) cells/µL in TN participants and by + 30 (−50 to 123) cells/µL in TE participants. Persistence on B/F/TAF was high through month 12 with 10% (1/10) of TN and 7 % (11/160) of TE participants discontinuing B/F/TAF within 12 months of initiation of treatment. No resistance to B/F/TAF emerged. Study drug-related adverse events occurred in 7% (12/169) of participants, leading to B/F/TAF discontinuation in 4 of 169 participants. Improvements in treatment satisfaction were observed in TE participants. B/F/TAF demonstrated high levels of effectiveness, persistence, and treatment satisfaction, and was well tolerated through month 12 in people with HIV treated in routine clinical practice in Canada.

## 1. Introduction

In 2020, there were an estimated 62,790 people with human immunodeficiency virus (HIV) in Canada,^[1]^ with another 1833 new cases diagnosed in 2022.^[2]^ Advances in antiretroviral therapy (ART) have improved mortality and morbidity rates,^[3,4]^ and there is a growing population of older people with HIV worldwide.^[5]^ At least 1 in 5 of people with new HIV diagnoses in Canada are aged ≥ 50 years.^[6]^ ART has become a life-long treatment. Factors driving long-term success in people with HIV include sustained undetectable viral load, minimal impact of treatment/monitoring, and optimized health-related quality of life.^[7]^ Optimal regimens need to demonstrate long-term virologic suppression, high tolerability, a favorable safety profile, a high resistance barrier, low potential for drug–drug interactions, and low pill burden.^[3,7,8]^

Simplifying treatment to reduce pill burden and the potential for drug–drug interactions is an important consideration for improving treatment adherence, persistence, and quality of life,^[3,7,9]^ particularly in older people who often have greater rates of comorbidity and polypharmacy.^[10,11]^ Therefore, in addition to virologic control, managing comorbidity and polypharmacy has become increasingly important for healthcare professionals providing care for people with HIV.

Bictegravir/emtricitabine/tenofovir alafenamide (B/F/TAF) is a 3-drug ART regimen co-formulated as a single tablet. Many clinical trials have demonstrated the safety and efficacy of B/F/TAF in ART-naïve (TN) and ART-experienced (TE) people with HIV,^[12–18]^ including older individuals with high levels of polypharmacy and comorbidity.^[13]^ Based on this supporting evidence, B/F/TAF is indicated for the treatment of HIV-1 in multiple countries, including Canada, where it was approved by Health Canada in July 2018. B/F/TAF is recommended as an initial first-line treatment for HIV in major HIV guidelines, including those from the US Department of Health and Human Services, the International Antiviral Society-USA, the European AIDS Clinical Society, Québec’s consultative committee on HIV and hepatitis C virus, and the British Columbia Centre for Excellence in HIV/AIDS.^[3,8,19–21]^

Several small real-world evidence (RWE) studies have shown the effectiveness and safety of B/F/TAF in routine clinical practice.^[22–32]^ However, there is little RWE with B/F/TAF in the Canadian population. The only available evidence from Canada comes from specific population groups, such as TN migrant people in a Montreal-based multidisciplinary HIV care clinic receiving rapid cost-covered B/F/TAF initiation,^[31,32]^ and a retrospective chart review in people with previously documented primary nucleoside reverse transcriptase inhibitor (NRTI) resistance at a single center in Alberta.^[30]^

BICSTaR (BICtegravir Single Tablet Regimen) is an ongoing, observational cohort study that has enrolled 2379 TN and TE participants from 14 countries across 5 cohorts. The study follows people from all countries for 24 months. The primary objective is to measure the effectiveness of B/F/TAF at month 12. Secondary/additional objectives include longitudinal analysis of virologic effectiveness and immunologic outcomes, safety/tolerability, persistence, and treatment satisfaction.

Pooled, prospective, 12-month data from 12 countries in BICSTaR have been published elsewhere.^[33]^ Here, we report 12-month effectiveness, safety, and tolerability data for 170 people with HIV in the BICSTaR Canada cohort.

## 2. Methods

### 2.1. Study design and setting

Detailed methods for the BICSTaR program are described elsewhere.^[33]^ In this analysis, we included individuals aged ≥ 18 years with HIV-1 in Canada and initiating B/F/TAF in routine clinical practice at 1 of 6 medical centers: Clinique de Médicine Urbaine du Quartier Latin, Montreal; Maple Leaf Research, Toronto; Regina General Hospital, Regina; St Clair Medical Associates, Toronto; Spectrum Health, Vancouver; and The Ottawa Hospital, Ottawa.

The data presented here were prospectively collected between November 14, 2018 and September 4, 2020 by trained personnel, and entered into standardized electronic Case Report Forms from clinical records, hospital files, clinic visits, electronic medical records, and validated questionnaires. Follow-up visits were conducted according to standard practice at each site, based on the decision of the treating physician. Adverse events (AEs) were coded using the Medical Dictionary for Regulatory Activities Version 24.1.

### 2.2. Participants and treatment

Eligible participants were adults with HIV-1 who provided written informed consent following the physician’s independent decision to treat with B/F/TAF. Participant eligibility criteria were well defined, and all consenting participants who met these criteria were included to reduce selection bias. Participants were treated with B/F/TAF (50 mg/200 mg/25 mg) in accordance with the Canadian product monograph.^[34]^ Data collection commenced from the participant’s enrollment in the program. The Canada cohort will be followed for 24 months in the main BICSTaR study, with an additional extension through 60 months. Participants discontinuing B/F/TAF could remain in the study and be followed up with the new ART regimen until premature end of documentation (e.g. lost to follow-up) or until the end of the main study (24 months after enrollment).

### 2.3. Study endpoints and assessments

Detailed endpoint and assessment information for the BICSTaR program are described elsewhere.^[33]^ Follow-up visits occurred according to the standard practice of each site and the judgment of the treating physician. No additional diagnostic or laboratory monitoring procedures were required.

The primary endpoint was the proportion of participants with HIV-1 RNA < 50 copies/mL at month 12. Secondary endpoints included the proportion of participants with HIV-1 RNA < 50 copies/mL at months 3 and 6, change from baseline in CD4 count and CD4/CD8 ratio at month 12, and the proportion of participants experiencing AEs and serious AEs (SAEs) at month 12. Additional exploratory endpoints included: reasons for initiating ART in TN participants, and reasons for switching to B/F/TAF in TE participants; weight and body mass index (BMI) at month 12; laboratory parameters, including metabolic assessments (total cholesterol, high-density lipoprotein, low-density lipoprotein, triglycerides) and renal function; proportion of participants discontinuing B/F/TAF within 12 months as an indicator of treatment persistence; reasons for discontinuation; and treatment satisfaction at baseline, month 6, and month 12. Treatment satisfaction was measured using the HIV Treatment Satisfaction status (HIVTSQs) and change (HIVTSQc) questionnaires.^[35]^ The score for the HIVTSQs questionnaire ranges from 0 to 60, and the score for the HIVTSQc questionnaire ranges from −30 to 30, with higher scores indicating improved satisfaction, lower scores indicating reduced satisfaction, and scores of 0 indicating no change in treatment satisfaction.

### 2.4. Statistical analysis

The analysis population included all participants enrolled in the study with sufficient follow-up time at a predefined date (September 4, 2020).

The primary endpoint analysis was B/F/TAF effectiveness (virologic suppression) at month 12, measured by a missing-equals-excluded (M = E) analysis in participants with ≥ 1 HIV-1 RNA value available within the 12-month time window (≥275 days [or 9 months] to ≤ 548 days [or 18 months]). If a participant’s viral load was measured on two or more occasions during the window, then the last measurement was used for the analysis, as per recommendations from the US Department of Health and Human Services Food and Drug Administration Center for Drug Evaluation and Research.^[36]^ Participants with missing data, or who discontinued the study and/or B/F/TAF before the 12-month window, were not included (no imputation). M = E analysis was also performed at months 3 and 6.

A treatment discontinuation-equals-failure (D = F) sensitivity analysis was performed for effectiveness at months 3, 6, and 12. At month 12, participants with ≥ 1 HIV-1 RNA value within the 12-month time window, and those who discontinued B/F/TAF before the start of the 12-month window (considered as treatment failures, and imputed as having HIV-1 RNA ≥ 50 copies/mL), were included.

Descriptive statistics were used for demographics and outcomes analyses, and reported for both the TN and TE populations. Changes from baseline to month 12 in CD4 count, CD4/CD8 ratio, weight, BMI, lipids, and estimated glomerular filtrate rate (eGFR) were assessed using 95% 2-sided *P* values and/or confidence intervals (CIs) and sign tests. Statistical testing was not performed when the sample size was < 20. The Cockcroft-Gault formula was used to calculate eGFR.^[37]^ Statistical analyses were performed using SAS software, version 9.4 (SAS Institute).

### 2.5. Ethics approval statement

The protocol was approved by the independent ethics committee at each center, and the study was conducted following Ethical Guidelines for Medical and Health Research Involving Human Subjects. All participants provided signed informed consent.

## 3. Results

### 3.1. Baseline demographics and disease characteristics

In total, 201 participants were enrolled in the BICSTaR Canada cohort from sites across Ontario, Saskatchewan, Quebec, and British Columbia, and 170 (TN, n = 10; TE, n = 160) were included in the analysis population at the time of data cutoff (September 04, 2020; see Figure S1, Supplementary Digital Content, http://links.lww.com/MD/M154). Median follow-up time was 17.8 months, and median duration of B/F/TAF treatment was 17.7 months.

Participants were predominantly male and White, with TE participants older, on average, than TN participants (Table 1). Most participants had at least 1 comorbidity at baseline (neuropsychiatric conditions being the most common), and most were receiving at least 1 concomitant, non-ART medication. Simplifying ART was the most common reason for switching to B/F/TAF from previous regimens (68%). In total, 11% (19/170) of participants (17 TE and 2 TN) had evidence of ≥ 1 preexisting primary resistance mutations (NRTI = 7%; non-nucleoside reverse transcriptase inhibitor [NNRTI] = 6%), with K103N/S (n = 7) and M184V/I (n = 6) mutations the most common (see Table S1, Supplementary Digital Content, http://links.lww.com/MD/M157).

**Table 1:** Baseline demographics and disease characteristics.

	All (n = 170)	TN (n = 10)	TE (n = 160)
Sex,* n (%)			
Male	150 (88.2)	10 (100)	140 (87.5)
Female	20 (11.8)	0	20 (12.5)
Age			
Median (Q1–Q3), yr	50.0 (39.0–58.0)	38.5 (28.0–53.0)	51.0 (41.0–58.0)
<50, n (%)	82 (48.2)	6 (60.0)	76 (47.5)
≥50, n (%)	88 (51.8)	4 (40.0)	84 (52.5)
Median (Q1–Q3) weight, kg	79.1 (69.2–91.4)	81.3 (78.9–91.8)	79.0 (69.2–91.4)
Median (Q1–Q3) BMI, kg/m^2^	26.3 (23.3–29.7)	26.2 (24.8–30.9)	26.3 (23.3–29.7)
Median (Q1–Q3) eGFR, mL/min/1.73 m^2^	98.45 (81.15–123.15)	116.45 (98.80–140.93)	97.86 (80.77–122.97)
Race/ethnicity, n (%)			
White	123 (72.4)	5 (50)	118 (73.8)
Black	19 (11.2)	2 (20.0)	17 (10.6)
Asian	12 (7.1)	2 (20.0)	10 (6.3)
American Indian/Alaska Native	1 (0.6)	0	1 (0.6)
Other	15 (8.8)	1 (10.0)	14 (8.8)
Comorbidities/co-infections, n (%)			
Any	153 (90.0)	8 (80.0)	145 (90.6)
None	17 (10.0)	2 (20.0)	15 (9.4)
1	26 (15.3)	0	26 (16.3)
2	19 (11.2)	2 (20.0)	17 (10.6)
≥3	108 (63.5)	6 (60.0)	102 (63.8)
Most common			
Neuropsychiatric	65 (38.2)	3 (30.0)	62 (38.8)
Hyperlipidemia	45 (26.5)	2 (20.0)	43 (26.9)
Hypertension	40 (23.5)	2 (20.0)	38 (23.8)
Osteopathic	33 (19.4)	1 (10.0)	32 (20.0)
Asthma†	22 (13.0)	2 (20.0)	20 (12.6)
Cardiovascular	19 (11.2)	1 (10.0)	18 (11.3)
HIV-1 RNA viral load‡			
Median (Q1–Q3), log_10_ copies/mL	1.59 (1.59–1.59)	4.88 (4.43–5.38)	1.59 (1.59–1.59)
<50 copies/mL, n (%)	141 (91.0)	1 (10.0)	140 (96.6)
>100,000 copies/mL, n (%)	4 (2.6)	4 (40.0)	0
Median (Q1–Q3) CD4 count, cells/µL§	564.0 (390.0–740.0)	391.5 (109.0–581.0)	586.0 (400.0–747.0)
Median (Q1–Q3) CD4/CD8 ratio‖	0.80 (0.59–1.20)	0.42 (0.10–0.80)	0.88 (0.60–1.23)
Late HIV diagnosis, n (%)			
CD4 <350 cells/µL¶	–	4 (40.0)	–
CD4 <200 cells/µL¶	–	3 (30.0)	–
Concomitant non-ART medications at baseline, n (%)#			
None	29 (17.3)	2 (22.2)	27 (17.0)
1	31 (18.4)	0	31 (19.5)
2	28 (16.7)	0	28 (17.6)
≥3	80 (47.6)	7 (77.8)	73 (45.9)
Median (Q1–Q3) number of previous ART regimens**	–	–	2.0 (1.0–4.0)
Prior ART regimens (taken just prior to B/F/TAF), n (%)††			
INSTI	–	–	109 (68.6)
NNRTI	–	–	36 (22.6)
PI	–	–	18 (11.3)
TDF	–	–	73 (45.9)
TAF	–	–	42 (26.4)
History of prior virologic failure, n (%)‡‡	–	–	3 (1.9)
Median (Q1–Q3) time from HIV diagnosis to B/F/TAF initiation, days	–	13.0 (7.0–79.0)	–

AIDS = acquired immunodeficiency syndrome, ART = antiretroviral treatment, B/F/TAF = bictegravir/emtricitabine/tenofovir alafenamide, BMI = body mass index, eGFR = estimated glomerular filtrate rate, HIV = human immunodeficiency virus, INSTI = integrase strand transfer inhibitor, NNRTI = non-nucleoside reverse transcriptase inhibitor, PI = protease inhibitor, Q = quartile, TAF = tenofovir alafenamide, TDF = tenofovir disoproxil fumarate, TE = treatment-experienced, TN = treatment-naïve.

* Sex was defined by the individual.

^†^Missing in 1 TE participant.

^‡^Sample size: TN (n = 10), TE (n = 145).

^§^Sample size: TN (n = 10), TE (n = 137).

^‖^Sample size: TN (n = 10), TE (n = 132).

^¶^And/or ≥ 1 AIDS-defining event at baseline.

^#^Missing in 1 TN and 1 TE participants.

** Sample size (n = 157).

^††^Missing in 1 participant, sample size (n = 159).

^‡‡^Unknown in 11 TE participants.

### 3.2. Effectiveness

**Figure 1.**
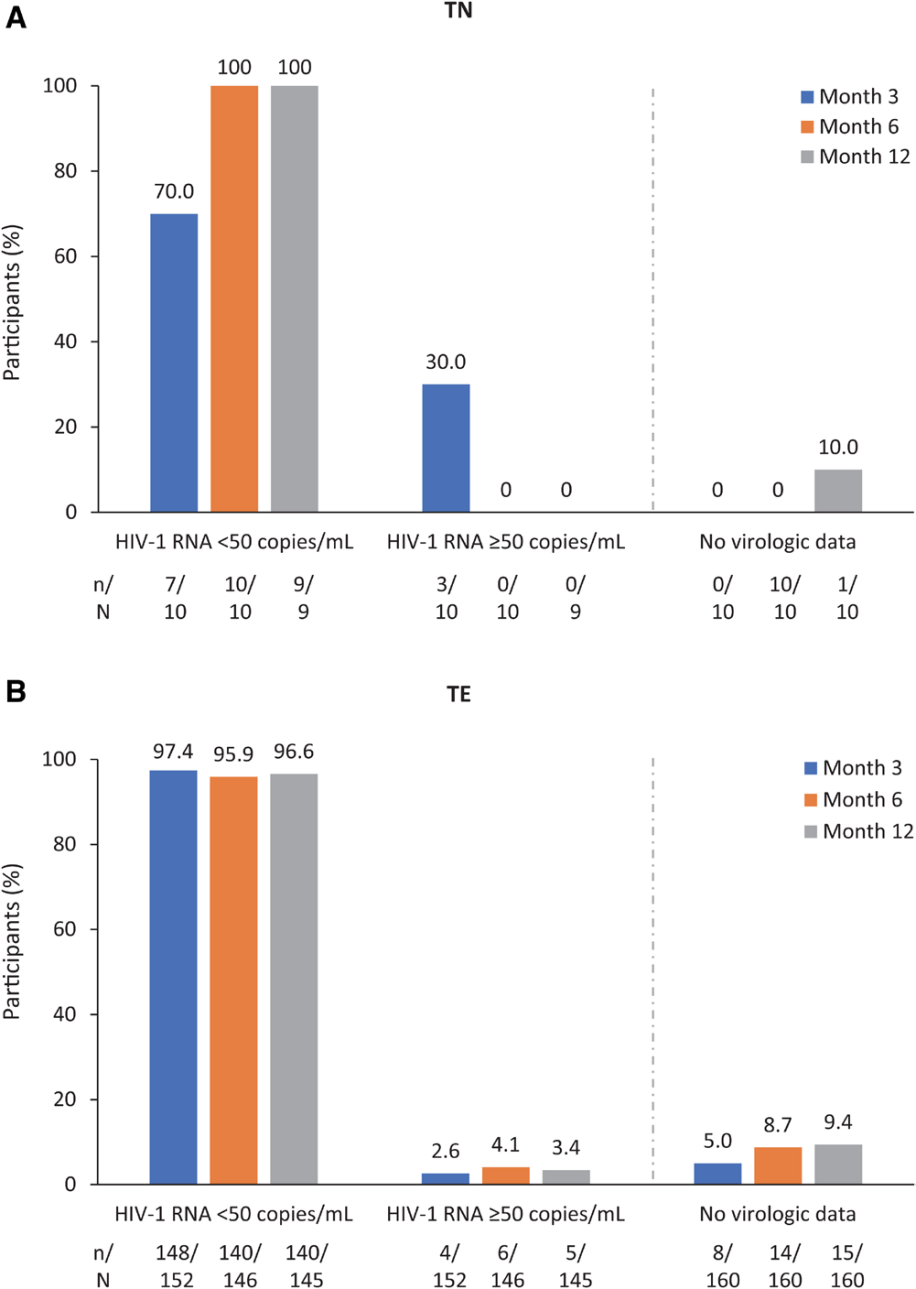
Virologic outcomes at months 3, 6, and 12 (M = E analysis) in (A) TN participants and (B) TE participants. Not all participants had virologic data available during each time window. Denominators reflect the number of participants with a value available and analyzed within the time window; only values available within each time window are analyzed. Participants who discontinued the study (and/or B/F/TAF) before the time window, and those still on the study and treated with B/F/TAF but without a HIV-1 RNA value available within the time window were, not considered (no imputation) for M = E analysis. Of the 5/145 TE participants with viral load ≥ 50 copies/mL at month 12, all 5 participants had HIV-1 RNA < 100 copies/mL. B/F/TAF = bictegravir/emtricitabine/tenofovir alafenamide, HIV = human immunodeficiency virus, M = E = missing-equals-excluded, TE = treatment-experienced, TN = treatment-naïve.

The primary endpoint (M = E) analysis showed that B/F/TAF achieved or maintained virologic suppression (HIV-1 RNA < 50 copies/mL) in 100% (9/9) of TN participants and 97% (140/145) of TE participants at month 12 (Figure 1; see explanation of denominators for M = E and D = F analysis at month 12 in Figure S1, Supplementary Digital Content, http://links.lww.com/MD/M154). In the D = F analysis, the proportions of participants with virologic suppression were 100% (9/9) and 93% (140/150) for TN and TE participants, respectively (see Table S2, Supplementary Digital Content, http://links.lww.com/MD/M158). Five participants (all in the TE group) had HIV-1 RNA ≥ 50 copies/mL at month 12; HIV-1 RNA was < 100 copies/mL in all 5 cases. B/F/TAF was discontinued in 1/5 of these participants (reason for discontinuation was a reported drug-related adverse event [DRAE] of weight gain). There were no recorded cases of treatment-emergent resistance through month 12.

Median CD4 cell count and CD4/CD8 ratios increased from baseline to month 12 in both TN and TE participants (Table 2). Median CD4 counts increased by + 195 cells/µL in TN participants and by + 30 cells/µL in TE participants (*P* < .05). Median CD4/CD8 ratios increased by + 0.2 in TN participants and by + 0.03 in TE participants (*P* < .01).

**Table 2:** CD4 cell count and CD4/CD8 ratio (and categories) through month 12.

	TN (n = 10)	TE (n = 160)
CD4 cell count		
n	10	137
Baseline, median (Q1–Q3) cells/µL	391.5 (109.0–581.0)	586.0 (400.0–747.0)
Change, median (Q1–Q3) cells/µL	+195.0 (124.5–307.0)	+30.0 (−50.0 to 123.0)
* P* value	NA	.047
CD4/CD8 ratio		
n	10	132
Baseline, median (Q1–Q3)	0.42 (0.10–0.80)	0.88 (0.60–1.23)
Change, median (Q1–Q3)	+0.2 (0.06–0.3)	+0.03 (−0.03 to 0.1)
* P* value	NA	.009
CD4/CD8 ratio (category)		
n	10	132
Baseline < 0.9, n (%)	8 (80.0)	68 (51.5)
Baseline 0.9–1.9, n (%)	2 (20.0)	59 (44.7)
Baseline > 1.9, n (%)	0	5 (3.8)
n	8	132
12 months, <0.9, n (%)	4 (50.0)	65 (49.2)
12 months, 0.9–1.9, n (%)	4 (50.0)	60 (45.5)
12 months, >1.9, n (%)	0	7 (5.3)

*P* values were calculated using the sign test for the null hypothesis that the median is equal to zero. *P* values were not calculated for TN participants due to the small sample size.

NA = not available, Q = quartile, TE = treatment-experienced, TN = treatment-naïve.

### 3.3. Persistence and study drug discontinuations

At month 12, persistence was high, with 10% (1/10) of TN participants and 7% (11/160) of TE participants discontinuing B/F/TAF within 12 months of initiation of treatment. One TN participant discontinued B/F/TAF due to subject decision. One participant in the TE group discontinued B/F/TAF after 8 months due to lack of efficacy (i.e., investigator-reported “LoE,” a pre-defined category in the Case Report Form). HIV-1 RNA was 222 copies/mL at month 8 and no emergent resistance was identified. The participant was switched to another ART (abacavir/dolutegravir/lamivudine). Other reasons for discontinuation in TE participants were death (n = 1), investigator discretion (n = 1), subject decision (n = 3), and AEs (n = 5).

### 3.4. Safety and tolerability

By month 12, 64% (109/170) of participants experienced at least 1 AE: 80% (8/10) of TN participants and 63% (101/160) of TE participants (Table 3). In total, 3% (5/170) of participants (all in the TE group) discontinued treatment due to an AE. There were no SAEs.

**Table 3:** Adverse events reported through month 12.

Summary of AEs, n (%)	All (n = 170)	TN (n = 10)	TE (n = 160)
Any AE	109 (64.1)	8 (80.0)	101 (63.1)
DRAEs*	12 (7.1)	0	12 (7.5)
Weight increased†	4 (2.4)	0	4 (2.5)
Psychiatric disorders‡	3 (1.8)	0	3 (1.9)
Headache	2 (1.2)	0	2 (1.3)
GERD	1 (0.6)	0	1 (0.6)
Herpes simplex	1 (0.6)	0	1 (0.6)
Alopecia	1 (0.6)	0	1 (0.6)
Any SAE*,§	0	0	0
Discontinued B/F/TAF due to DRAEs	4 (2.4)	0	4 (2.5)
Deaths‖	1 (0.6)	0	1 (0.6)

AE = adverse event, B/F/TAF = bictegravir/emtricitabine/tenofovir alafenamide, BMI = body mass index, DRAE = drug-related adverse event, GERD = gastroesophageal reflux disease, Q = quartile, SAE = serious adverse event, TE = treatment-experienced, TN = treatment-naïve.

* Missing in 1 TE participant.

^†^Median (Q1–Q3) baseline body weight and BMI for the 4 participants reporting increased weight were 83.8 kg (79.5–87.3) and 27.1 kg/m^2^ (24.1–29.9), respectively. Body weight and BMI data were available at 12 months for 2 participants (median [Q1–Q3] change: +5.5 kg [4.4–6.6] and +1.8 kg/m^2^ [1.5–2.2], respectively).

^‡^Abnormal dreams (n = 1; unknown psychiatric disorder at baseline but not taking psychiatric drugs), aggravated anxiety (n = 1; anxiety at baseline and on an antiepileptic agent), and major depression (n = 1; history of psychiatric problems that were not ongoing at baseline; the participant started treatment with escitalopram around the time of the AE).

^§^Seriousness criteria were: led to death, life threatening, initial/prolonged hospitalization, persistent or significant disability, or other significant medical event.

^‖^Primary cause of death was unknown, but not considered related to B/F/TAF.

DRAEs occurred in 7% (12/169) of participants (all in the TE group); all were mild-to-moderate, with the most common being weight increase (n = 4 participants) and psychiatric disorders (n = 3 participants). Median (quartile [Q]1–Q3) change in body weight for participants reporting a DRAE of weight increase was + 5.5 kg (4.4–6.6) at month 12, among 2 participants with available data at baseline and month 12 (see Table 3 for details). Among the 4 participants reporting a DRAE of weight increase, 3 had taken tenofovir disoproxil fumarate (TDF) prior to switching to B/F/TAF; 1 of these had also used a co-medication associated with weight gain (escitalopram) during the treatment period. Among the 3 participants reporting a psychiatric disorder DRAE, 2 had psychiatric disorders at baseline and 1 had a history of psychiatric problems that were not ongoing at baseline. Four participants discontinued B/F/TAF as a result of a DRAE: 1 participant had experienced moderate worsening of anxiety, and another participant had experienced mild, intermittent headache (both DRAEs resolved following drug withdrawal), and 2 participants experienced moderate weight increase. There were no B/F/TAF discontinuations due to hepatic, renal, or bone DRAEs. One TE participant died (primary cause of death unknown) during the 12-month study period; the death was not considered B/F/TAF-related (Table 3).

In the population with weight and BMI data available at baseline and month 12, TN and TE participants gained a median of +1.6 (0.5–4.7) kg and + 0.7 (−1.3 to 2.7) kg, respectively, through month 12 (Table 4). Median (Q1–Q3) baseline BMI was 26.2 (22.3–31.0) kg/m^2^ in TN participants and 26.4 (23.8–29.8) kg/m^2^ in TE participants, and increased slightly at month 12 (+0.6 [0.1–1.4] and +0.2 [−0.5 to 0.8], respectively) (Table 4). Changes in lipid profiles were minimal at month 12 (see Figure S2, Supplementary Digital Content, http://links.lww.com/MD/M155). Median (Q1–Q3) baseline eGFR was 116.45 (98.80–140.93) mL/min/1.73 m^2^ in TN participants and 97.86 (80.77–122.97) mL/min/1.73 m^2^ in TE participants. Small median reductions in eGFR were observed from baseline to month 12 in the TN (−16.11 mL/min/1.73 m^2^) and TE (−3.62 mL/min/1.73 m^2^) groups (see Figure S3, Supplementary Digital Content, http://links.lww.com/MD/M156).

**Table 4:** Weight and body mass index analyses at month 12.*

	TN (n = 7)*	TE (n = 117)*
Weight		
Median baseline (Q1–Q3), kg	81.3 (78.9–95.0)	79.0 (70.0–91.3)
Min–Max	62.5–98.9	47.9–153.0
Change from baseline		
Median (Q1–Q3), kg	+1.6 (0.5–4.7)	+0.7 (−1.3 to 2.7)
* P* value for change from baseline	NA	0.05073
Min–Max	−10.3 to +6.7	−12.6 to +10.7
<10% weight gain, n (%)	1 (14.3)	4 (3.3)
>10% weight loss, n (%)	0	3 (2.5)
BMI		
Median baseline (Q1–Q3), kg/m^2^	26.2 (22.3–31.0)	26.4 (23.8–29.8)
Min–Max	19.2–33.1	18.5–42.8
Change from baseline		
Median (Q1–Q3), kg/m^2^	+0.6 (0.1–1.4)	+0.2 (−0.5 to 0.8)
* P* value for change from baseline	NA	0.02785
Min–Max	−3.6 to 2.1	−4.5 to 3.0
Categorical changes		
Underweight (<18.5 kg/m^2^), n (%)		
Baseline	0	0
Month 12	0	0
Normal (18.5–24.9 kg/m^2^), n (%)		
Baseline	2 (28.6)	42 (35.9)
Month 12	2 (28.6)	38 (32.5)
Overweight (25.0–29.9 kg/m^2^), n (%)		
Baseline	2 (28.6)	47 (40.2)
Month 12	3 (42.9)	50 (42.7)
Obese (≥30 kg/m^2^), n (%)		
Baseline	3 (42.9)	28 (23.9)
Month 12	2 (28.6)	29 (24.8)

*P* values were calculated using the sign test for the null hypothesis that the median is equal to zero.

BMI = body mass index, NA = not available, Q = quartile, TE = treatment-experienced, TN = treatment-naïve.

* Restricted population in participants with weight and BMI data available at baseline and month 12 (n = 124).

### 3.5. Treatment satisfaction

The number of TN participants completing the HIVTSQs at baseline (n = 10) and the HIVTSQc at month 6 (n = 10) and month 12 (n = 9) was small, prohibiting meaningful analysis of treatment satisfaction among this group. In the TE group, based on HIVTSQs score at baseline (n = 134), satisfaction with current ART was high. Following the switch to B/F/TAF, improvements in treatment satisfaction were observed at months 6 and 12, with a median (Q1–Q3) total change (HIVTSQc) score of + 24.0 (17.0–30.0) at month 6 (n = 150) and + 25.0 (14.5–30.0) at month 12 (n = 137), where a score of + 30 represents the maximum for increased treatment satisfaction (Table 5).

**Table 5:** Treatment satisfaction at baseline and change from baseline at months 6 and 12 in treatment-experienced participants.*

	TE (n = 160)
Treatment satisfaction total score	
Baseline (HIVTSQs)	
n	134
Median (Q1–Q3) total score	54.0 (48.0–60.0)
Month 6 (HIVTSQc)	
n	150
Median (Q1–Q3) change from baseline	+24.0 (17.0–30.0)
Month 12 (HIVTSQc)	
n	137
Median (Q1–Q3) change from baseline	+25.0 (14.5–30.0)

HIVTSQs/c = HIV Treatment Satisfaction Questionnaire Status/Change, Q = quartile, TE = treatment-experienced.

* Participants with score available at baseline and month 12.

## 4. Discussion

Here, we report the 12-month effectiveness, safety, tolerability, persistence, and satisfaction with B/F/TAF treatment in routine clinical practice in the Canadian BICSTaR cohort of people with HIV.

B/F/TAF achieved high levels of virologic suppression (>96%; M = E analysis) at month 12 amongst TN and TE participants, demonstrating the effectiveness of the treatment and supporting the findings of various clinical studies.^[12–18]^ CD4 cell count and CD4/CD8 ratio increased from baseline in both groups at month 12. The small number of TN participants precluded statistical testing in this group, but statistically significant improvements in CD4 count (*P < *.05) and CD4/CD8 (*P < *.01) ratio were observed in TE participants, reflecting the benefits of B/F/TAF on immune restoration noted in clinical trials.^[15,18]^ No new safety concerns were identified. The proportion of people reporting DRAEs was low and there were no SAEs. The safety and tolerability findings in this real-world cohort are consistent with the known profile of B/F/TAF from clinical studies,^[13,18]^ and are reflected in low rates of discontinuation and therefore high persistence.

TN and TE participants gained a median of + 1.6 (0.5–4.7) kg and + 0.7 (−1.3 to 2.7) kg, respectively. The median weight gain for TE participants was consistent with the typical weight gains of 0.5 to 1 kg per year reported in Western adult populations.^[38]^ ART-associated weight gain in TN people with HIV may partly be due to a return to health with weight returning to pre-HIV infection levels.^[39]^ Furthermore, some ART drugs (e.g., TDF) are associated with weight suppressant effects that may increase the risk of weight gain following a switch to an alternative ART regimen.^[40]^ Notably, 3 of the 4 participants reporting a DRAE of weight gain on B/F/TAF had switched from prior regimens containing efavirenz and TDF.

Until now, there has been little RWE with B/F/TAF, specifically in the Canadian population. One small qualitative study conducted in Montreal showed the value of rapid cost-covered B/F/TAF initiation in 16 migrant people with HIV.^[31]^ Most individuals indicated that they were satisfied with treatment at initiation, mainly due to lack of side effects.^[31]^ Ongoing treatment adherence was also linked to treatment satisfaction, with many attributing this to improved health and ease of treatment.^[31]^ Another analysis from the same study in 35 migrant people with HIV identified social determinants associated with a longer timeframe to initiate B/F/TAF; none of these factors had a statistically significant impact on the time to achieve viral suppression (HIV viral load of < 50 copies/mL).^[32]^ A retrospective chart review conducted at the University of Alberta, Edmonton, confirmed the efficacy of B/F/TAF in 50 people with previously documented primary NRTI resistance.^[30]^ Our study provides the first prospective RWE for B/F/TAF treatment in a broad range of people with HIV across Canada.

A recent observational study in 19,322 people with HIV in Canada suggests that adherence to ART may be suboptimal in many (44.7%), and adherence is lower for those receiving multi-tablet versus single-tablet regimens.^[41]^ Simplifying HIV treatment is an important consideration, particularly in older people who often have higher rates of comorbidity and polypharmacy.^[10,11]^ As the population of older people with HIV is growing worldwide,^[5]^ evaluating the real-world effectiveness and safety of single-tablet regimens in this context is becoming increasingly important. It is notable that in our Canadian cohort, effectiveness and safety of B/F/TAF was demonstrated in an analysis population that included 51.8% of people ≥ 50 years of age, 63.5% with ≥ 3 comorbidities and 47.6% with ≥ 3 concomitant medications.

The use of a common protocol across all BICSTaR cohorts allows for direct comparison of these Canadian cohort data with the findings of larger, more diverse global populations in the BICSTaR program. Our findings are consistent with a pooled 12-month analysis of BICSTaR data from more than 1500 participants across 12 countries (including Canada),^[33]^ in which reported virologic suppression rates were 94% in TN participants and 97% in TE participants. Further analyses in the pooled cohort showed high B/F/TAF effectiveness in different groups of people with HIV, including in participants aged ≥ 50 years and in those with comorbidities at baseline.^[33]^ Data from both our Canadian cohort and the larger pooled cohort indicate immunologic restoration, favorable safety and tolerability, and high levels of persistence (low proportion of B/F/TAF discontinuation [<10%], including discontinuation due to DRAEs [<7%]) across both TN and TE participants at month 12 follow-up in both studies.

There are limitations to our analyses, such as lack of randomization, and selection and information bias, which are inherent to observational cohort studies. To reduce these effects, eligibility criteria were clearly defined, and standard measurement instruments and appropriate personnel training were used for data entry. The number of participants was relatively small; there were only 10 TN participants, preventing meaningful interpretation of findings in this group. A proportion of data was also missing, potentially due to unprecedented disruption at the start of the coronavirus disease pandemic leading to missed clinic visits. Nevertheless, our study was part of a larger multicenter program, and our results are consistent with those of the BICSTaR pooled analysis in more than 1500 participants from 12 countries,^[33]^ lending support to the findings in this Canada cohort.

## 5. Conclusion

Over 12 months, B/F/TAF demonstrated high levels of effectiveness and persistence among people with HIV in routine clinical practice across four Canadian provinces, many of whom had chronic comorbidities and were taking multiple concomitant medications. B/F/TAF was well tolerated, had a favorable safety profile, and no new safety signals were detected in this real-world setting. TE participants reported further improvements in already high treatment satisfaction rates after switching to B/F/TAF. Overall, our findings support the ongoing use of B/F/TAF for people with HIV living in Canada.

## Acknowledgments

Medical writing support, including development of a draft outline and subsequent drafts in consultation with the authors, collating author comments, copyediting, fact checking, and referencing, was provided by Josh Lilly, PhD at Aspire Scientific Limited (Bollington, UK). Funding for medical writing support for this article was provided by Gilead Sciences Europe Ltd (Uxbridge, UK). The sponsor, Gilead Sciences, designed the study and played a role in the data collection.

## Author contributions

**Conceptualization:** Andrea Marongiu, David Thorpe.

**Formal analysis:** Taban Saifi, Dylana Mumm, Andrea Marongiu, Rebecca Harrison, David Thorpe.

**Investigation:** Alexander Wong, Jason Brunetta, Joss De Wet, Ken Logue, Hugues Loemba, Taban Saifi, Dylana Mumm, Andrea Marongiu, Rebecca Harrison, David Thorpe, Benoit Trottier.

**Methodology:** Andrea Marongiu, Rebecca Harrison, David Thorpe.

**Project administration:** Taban Saifi, Dylana Mumm, Andrea Marongiu, Rebecca Harrison, David Thorpe.

**Resources:** Alexander Wong, Jason Brunetta, Joss De Wet, Ken Logue, Hugues Loemba, Taban Saifi, Dylana Mumm, Andrea Marongiu, Rebecca Harrison, David Thorpe, Benoit Trottier.

**Supervision:** Taban Saifi, Dylana Mumm, Andrea Marongiu, Rebecca Harrison, David Thorpe.

**Writing – original draft:** Alexander Wong, Jason Brunetta, Joss De Wet, Ken Logue, Hugues Loemba, Taban Saifi, Dylana Mumm, Andrea Marongiu, Rebecca Harrison, David Thorpe, Benoit Trottier.

**Writing – review & editing:** Alexander Wong, Jason Brunetta, Joss De Wet, Ken Logue, Hugues Loemba, Taban Saifi, Dylana Mumm, Andrea Marongiu, Rebecca Harrison, David Thorpe, Benoit Trottier.

## Supplementary Material











## References

[1] Public Health Agency of Canada. Estimates of HIV incidence, prevalence and Canada’s progress on meeting the 90-90-90 HIV targets 2020. 2022. Available at: https://www.canada.ca/en/public-health/services/publications/diseases-conditions/estimates-hiv-incidence-prevalence-canada-meeting-90-90-90-targets-2020.html [access date February 27, 2024].

[2] Public Health Agency of Canada. HIV in Canada: 2022 surveillance highlights. Available at: https://www.canada.ca/en/public-health/services/publications/diseases-conditions/hiv-2022-surveillance-highlights.html [access date February 27, 2024].

[3] Gandhi RT Bedimo R Hoy JF. Antiretroviral drugs for treatment and prevention of HIV infection in adults: 2022 recommendations of the International Antiviral Society-USA Panel. JAMA. 2023;329:63–84. 36454551 10.1001/jama.2022.22246

[4] Marcus JL Leyden WA Alexeeff SE. Comparison of overall and comorbidity-free life expectancy between insured adults with and without HIV infection, 2000-2016. JAMA Netw Open. 2020;3:e207954. 32539152 10.1001/jamanetworkopen.2020.7954 PMC7296391

[5] The Lancet Healthy Longevity. Ageing with HIV. Lancet Healthy Longev. 2022;3:e119. 36098283 10.1016/S2666-7568(22)00041-1

[6] Public Health Agency of Canada. HIV in Canada: 2021 surveillance highlights. Available at: https://www.canada.ca/en/public-health/services/publications/diseases-conditions/hiv-2021-surveillance-highlights.html [access date February 27, 2024].

[7] Lazarus JV Wohl DA Cascio M. Long-term success for people living with HIV: a framework to guide practice. HIV Med. 2023;24(Suppl 2):8–19. 36920412 10.1111/hiv.13460

[8] Panel on Antiretroviral Guidelines for Adults and Adolescents. Guidelines for the use of antiretroviral agents in adults and adolescents with HIV. 2022. Available at: https://clinicalinfo.hiv.gov/sites/default/files/guidelines/documents/adult-adolescent-arv/guidelines-adult-adolescent-arv.pdf [access date February 27, 2024].

[9] Nachega JB Parienti JJ Uthman OA. Lower pill burden and once-daily antiretroviral treatment regimens for HIV infection: a meta-analysis of randomized controlled trials. Clin Infect Dis. 2014;58:1297–307. 24457345 10.1093/cid/ciu046 PMC3982838

[10] Pelchen-Matthews A Ryom L Borges AH.; EuroSIDA study. Aging and the evolution of comorbidities among HIV-positive individuals in a European cohort. AIDS. 2018;32:2405–16. 30134296 10.1097/QAD.0000000000001967

[11] Marin S Quiñones C Codina-Jiménez C. The management of polypharmacy in people living with HIV. AIDS Rev. 2023;25:27–40. 36952662 10.24875/AIDSRev.M23000059

[12] Kityo C Hagins D Koenig E. Switching to fixed-dose bictegravir, emtricitabine, and tenofovir alafenamide (B/F/TAF) in virologically suppressed HIV-1 infected women: a randomized, open-label, multicenter, active-controlled, phase 3, noninferiority trial. J Acquir Immune Defic Syndr. 2019;82:321–8. 31609930 10.1097/QAI.0000000000002137

[13] Maggiolo F Rizzardini G Molina JM. Bictegravir/emtricitabine/tenofovir alafenamide in virologically suppressed people with HIV aged ≥65 years: Week 48 results of a phase 3b, open-label trial. Infect Dis Ther. 2021;10:775–88. 33686573 10.1007/s40121-021-00419-5 PMC8116430

[14] Molina JM Ward D Brar I. Switching to fixed-dose bictegravir, emtricitabine, and tenofovir alafenamide from dolutegravir plus abacavir and lamivudine in virologically suppressed adults with HIV-1: 48 week results of a randomised, double-blind, multicentre, active-controlled, phase 3, non-inferiority trial. Lancet HIV. 2018;5:e357–65. 29925489 10.1016/S2352-3018(18)30092-4

[15] Orkin C DeJesus E Sax PE.; GS-US-380-1489; GS-US-380-1490 Study Investigators. Fixed-dose combination bictegravir, emtricitabine, and tenofovir alafenamide versus dolutegravir-containing regimens for initial treatment of HIV-1 infection: week 144 results from two randomised, double-blind, multicentre, phase 3, non-inferiority trials. Lancet HIV. 2020;7:e389–400. 32504574 10.1016/S2352-3018(20)30099-0

[16] Sax PE Rockstroh JK Luetkemeyer AF.; GS-US-380–4030 Investigators. Switching to bictegravir, emtricitabine, and tenofovir alafenamide in virologically suppressed adults with human immunodeficiency virus. Clin Infect Dis. 2021;73:e485–93. 32668455 10.1093/cid/ciaa988 PMC8282313

[17] Stellbrink HJ Arribas JR Stephens JL. Co-formulated bictegravir, emtricitabine, and tenofovir alafenamide versus dolutegravir with emtricitabine and tenofovir alafenamide for initial treatment of HIV-1 infection: week 96 results from a randomised, double-blind, multicentre, phase 3, non-inferiority trial. Lancet HIV. 2019;6:e364–72. 31068272 10.1016/S2352-3018(19)30080-3

[18] Wohl DA Yazdanpanah Y Baumgarten A. Bictegravir combined with emtricitabine and tenofovir alafenamide versus dolutegravir, abacavir, and lamivudine for initial treatment of HIV-1 infection: week 96 results from a randomised, double-blind, multicentre, phase 3, non-inferiority trial. Lancet HIV. 2019;6:e355–63. 31068270 10.1016/S2352-3018(19)30077-3

[19] European AIDS Clinical Society (EACS). EACS guidelines. v12.0. 2023. Available at: https://www.eacsociety.org/guidelines/eacs-guidelines/ [access date February 27, 2024].

[20] British Columbia Centre for Excellence in HIV/AIDS. Therapeutic guidelines for antiretroviral (ARV) treatment of adult HIV infection. 2020. Available at: https://bccfe.ca/therapeutic-guidelines/guidelines-antiretroviral-arv-treatment-adult-hiv-infection [access date February 27, 2024].

[21] Gouvernement du Québec. La thérapie antirétrovirale pour les adultes infectés par le VIH. 2022. Available at: https://publications.msss.gouv.qc.ca/msss/fichiers/2022/22-337-01W.pdf [access date February 27, 2024].

[22] Ambrosioni J Rojas Lievano J Berrocal L. Real-life experience with bictegravir/emtricitabine/tenofovir alafenamide in a large reference clinical centre. J Antimicrob Chemother. 2022;77:1133–9. 35040990 10.1093/jac/dkab481

[23] Armenia D Forbici F Bertoli A. Bictegravir/emtricitabine/tenofovir alafenamide ensures high rates of virological suppression maintenance despite previous resistance in PLWH who optimize treatment in clinical practice. J Glob Antimicrob Resist. 2022;30:326–34. 35793776 10.1016/j.jgar.2022.06.027

[24] Chang HM Chou PY Chou CH. Outcomes after switching to BIC/FTC/TAF in patients with virological failure to protease inhibitors or non-nucleoside reverse transcriptase inhibitors: a real-world cohort study. Infect Drug Resist. 2021;14:4877–86. 34853517 10.2147/IDR.S331647 PMC8628065

[25] Lazzaro A Cacciola EG Borrazzo C. Switching to a bictegravir single tablet regimen in elderly people living with HIV-1: data analysis from the BICTEL cohort. Diagnostics (Basel). 2021;12:76. 35054243 10.3390/diagnostics12010076 PMC8774414

[26] Mican R de Gea Grela A Cadinanos J. Impact of preexisting nucleos(t)ide reverse transcriptase inhibitor resistance on the effectiveness of bictegravir/emtricitabine/tenofovir alafenamide in treatment experience patients. AIDS. 2022;36:1941–7. 35848506 10.1097/QAD.0000000000003311 PMC9612675

[27] Mounzer K Brunet L Fusco JS. Advanced HIV infection in treatment-naive individuals: effectiveness and persistence of recommended 3-drug regimens. Open Forum Infect Dis. 2022;9:ofac018. 35169590 10.1093/ofid/ofac018 PMC8842315

[28] Rolle CP Nguyen V Patel K. Real-world efficacy and safety of switching to bictegravir/emtricitabine/tenofovir alafenamide in older people living with HIV. Medicine (Baltimore). 2021;100:e27330. 34559154 10.1097/MD.0000000000027330 PMC8462546

[29] Sandulescu O Irimia M Benea OE. Treatment initiation or switch to BIC/FTC/TAF – real-world safety and efficacy data from two HIV centers in Romania. Germs. 2021;11:512–22. 35096668 10.18683/germs.2021.1286 PMC8789359

[30] Shafran SD Hughes CA. Bictegravir/emtricitabine/tenofovir alafenamide in patients with genotypic NRTI resistance. HIV Med. 2022;24:361–5. 35973753 10.1111/hiv.13376

[31] Arora AK Engler K Lessard D. Experiences of migrant people living with HIV in a multidisciplinary HIV care setting with rapid B/F/TAF initiation and cost-covered treatment: the ‘ASAP’ study. J Pers Med. 2022;12:1497. 36143282 10.3390/jpm12091497 PMC9503330

[32] Arora AK Vicente S Engler K. Impact of social determinants of health on time to antiretroviral therapy initiation and HIV viral undetectability for migrants enrolled in a multidisciplinary HIV clinic with rapid, free, and onsite B/F/TAF: ‘The ASAP study. HIV Med. 2024. [online ahead of print]. doi: 10.1111/hiv.13608. 10.1111/hiv.13608 38213087

[33] Esser S Brunetta J Inciarte A. Twelve-month effectiveness and safety of bictegravir/emtricitabine/tenofovir alafenamide in people with HIV: real-world insights from BICSTaR cohorts. HIV Med. 2023. [online ahead of print]. doi: 10.1111/hiv.13593. 10.1111/hiv.13593 38148567

[34] Gilead Sciences. BIKTARVY™ (bictegravir/emtricitabine/tenofovir alafenamide) tablets: Canadian product monograph. 2018. Available at: https://pdf.hres.ca/dpd_pm/00046296.PDF [access date February 27, 2024].

[35] Woodcock A Bradley C. Validation of the revised 10-item HIV Treatment Satisfaction Questionnaire status version and new change version. Value Health. 2006;9:320–33. 16961550 10.1111/j.1524-4733.2006.00121.x

[36] US Department of Health and Human Services, Food and Drug Administration Center for Drug Evaluation and Research. Human immonodeficiency virus-1 infection: developing antiretroviral drugs for treatment. Guidance for Industry. 2015. Available at: https://www.fda.gov/files/drugs/published/Human-Immunodeficiency-Virus-1-Infection--Developing-Antiretroviral-Drugs-for-Treatment.pdf [access date February 27, 2024].

[37] Cockcroft DW Gault MH. Prediction of creatinine clearance from serum creatinine. Nephron. 1976;16:31–41. 1244564 10.1159/000180580

[38] Hutfless S Maruthur NM Wilson RF. Strategies to Prevent Weight Gain Among Adults. Comparative Effectiveness Reviews, No. 97. Rockville, MD: Agency for Healthcare Research and Quality (US); 2013. 23638485

[39] Sax PE Erlandson KM Lake JE. Weight gain following initiation of antiretroviral therapy: risk factors in randomized comparative clinical trials. Clin Infect Dis. 2020;71:1379–89. 31606734 10.1093/cid/ciz999 PMC7486849

[40] Erlandson KM Carter CC Melbourne K. Weight change following antiretroviral therapy switch in people with viral suppression: pooled data from randomized clinical trials. Clin Infect Dis. 2021;73:1440–51. 33987636 10.1093/cid/ciab444 PMC12097995

[41] Angel JB Freilich J Arthurs E. Adherence to oral antiretroviral therapy in Canada, 2010-2020: a retrospective analysis of claims data. AIDS. 2023;37:2031–40. 37418513 10.1097/QAD.0000000000003648 PMC10552836

