# Supplementary material for: Twelve-month effectiveness and safety of bictegravir/emtricitabine/tenofovir alafenamide in people with HIV from the Canadian cohort of the observational BICSTaR study
Source: Medicine (Baltimore). 2024 Apr 19;103(16):e37785. doi: 10.1097/MD.0000000000037785 (PMC11029942; doi:10.1097/MD.0000000000037785)
Supplement: Supplementary file 1 [file medi-103-e37785-s001.docx]

**Supplementary Digital Content Figure** **1.** Participant flow diagram.


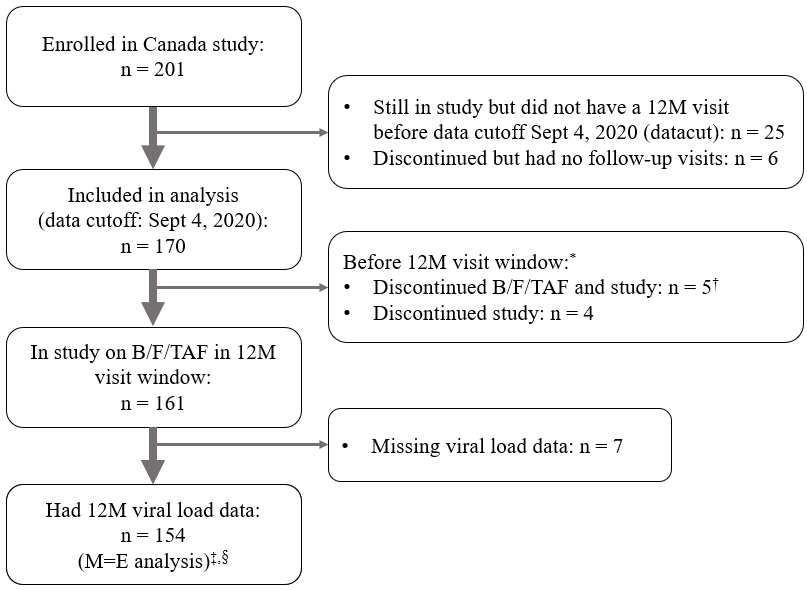


### *The 12M visit window was between 9 and 18 months. ^†^Participants included in D=F analysis (imputed as having HIV-1 RNA ≥50 copies/mL). ^‡^n = 159 participants included in D = F analysis. ^§^A further 3 participants discontinued B/F/TAF and the study during the 12M visit window but had 12M viral load data available before discontinuing and so were included in the effectiveness analysis. In total, 12 participants discontinued B/F/TAF and the study (n = 5 discontinued before the 12M visit window; n = 7 discontinued during the 12M visit window, with 4 of these having missing viral load data).

### 12M, 12 month; B/F/TAF = bictegravir/emtricitabine/ tenofovir alafenamide, D=F = discontinuation-equals-failure, M=E = missing-equals-excluded.
