# Supplementary material for: Twelve-month effectiveness and safety of bictegravir/emtricitabine/tenofovir alafenamide in people with HIV from the Canadian cohort of the observational BICSTaR study
Source: Medicine (Baltimore). 2024 Apr 19;103(16):e37785. doi: 10.1097/MD.0000000000037785 (PMC11029942; doi:10.1097/MD.0000000000037785)
Supplement: Supplementary file 2 [file medi-103-e37785-s002.docx]

## Supplementary Digital Content Table 1. Prevalence of key primary HIV drug-resistance mutations at baseline.

|  | **All (n = 170)** | **TN (n = 10)** | **TE (n = 160)** |
| --- | --- | --- | --- |
| ≥1 primary resistance mutation, n (%)* | | | |
| Yes | 19 (11.2) | 2 (20.0) | 17 (10.6) |
| No | 57 (33.5) | 5 (50.0) | 52 (32.5) |
| Primary resistance mutations of interest, n (%) | | | |
| NNRTI | 10 (5.9) | 2 (20.0) | 8 (5.0) |
| K103N/S | 7 (4.1) | 2 (20.0) | 5 (3.1) |
| PI | 0 (0) | 0 (0) | 0 (0) |
| NRTI | 12 (7.1) | 0 (0) | 12 (7.5) |
| M41L | 4 (2.4) | 0 | 4 (2.5) |
| K65R | 1 (0.6) | 0 | 1 (0.6) |
| D67N | 2 (1.2) | 0 | 2 (1.3) |
| M184V/I | 6 (3.5) | 0 | 6 (3.8) |
| K219Q/E/N/R | 2 (1.2) | 0 | 2 (1.3) |
| INSTI | 0 (0) | 0 (0) | 0 (0) |

*Genotype data were available for 76 participants at baseline (obtained either at the time of enrolment or from historic HIV-1 genotype tests); data were unavailable for n = 94
(TN, n = 3; TE, n = 91) – these participants were not considered to have primary resistance mutations.

INSTI = integrase strand transfer inhibitor, NNRTI = non-nucleoside reverse transcriptase inhibitor, NRTI = nucleoside reverse transcriptase inhibitor, PI = protease inhibitor,
TE = treatment-experienced, TN = treatment-naïve.
