# Supplementary material for: Twelve-month effectiveness and safety of bictegravir/emtricitabine/tenofovir alafenamide in people with HIV from the Canadian cohort of the observational BICSTaR study
Source: Medicine (Baltimore). 2024 Apr 19;103(16):e37785. doi: 10.1097/MD.0000000000037785 (PMC11029942; doi:10.1097/MD.0000000000037785)
Supplement: Supplementary file 3 [file medi-103-e37785-s003.docx]

## Supplementary Digital Content Table 2. Effectiveness at Months 3, 6, and 12 (D=F analysis).

|  | **TN**  **(n = 10)** | **TE (n = 160)** |
| --- | --- | --- |
| Baseline, n (%) | 10 (100) | 145 (90.6) |
| HIV-1 RNA viral load |  |  |
| <50 copies/mL | 1 (10.0) | 140 (96.6) |
| 95% CI | (0.3–44.5) | (92.1–98.9) |
| **≥**50 copies/mL | 9 (90.0) | 5 (3.4) |
| 95% CI | (55.5–99.7) | (1.1–7.9) |
| Month 3, n (%) | 10 (100) | 153 (95.6) |
| HIV-1 RNA viral load |  |  |
| <50 copies/mL | 7 (70.0) | 148 (96.7) |
| 95% CI | (34.8–93.3) | (92.5–98.9) |
| **≥**50 copies/mL | 3 (30.0) | 5 (3.3) |
| 95% CI | (6.7–65.2) | (1.1–7.5) |
| Month 6, n (%) | 10 (100) | 149 (93.1) |
| HIV-1 RNA viral load |  |  |
| <50 copies/mL | 10 (100) | 140 (94.0) |
| 95% CI | (69.2–100) | (88.8–97.2) |
| **≥**50 copies/mL | 0 | 9 (6.0) |
| 95% CI | - | (2.8–11.2) |
| Month 12, n (%) | 9 (90.0) | 150 (93.8) |
| HIV-1 RNA viral load |  |  |
| <50 copies/mL | 9 (100) | 140 (93.3) |
| 95% CI | (66.4–100) | (88.1–96.8) |
| **≥**50 copies/mL | 0 | 10 (6.7) |
| 95% CI | - | (3.2–11.9) |

At baseline, denominators reflect the number of participants with a value available and analyzed within the baseline time window (no imputation). At follow-up time windows, denominators reflect the number of participants with a value analyzed within the considered time window or who discontinued B/F/TAF before the considered time window and have been classified in the ≥50 copies/mL category (imputation for participants who discontinued before each time window).

B/F/TAF = bictegravir/emtricitabine/tenofovir alafenamide, CI = confidence interval,
D=F = discontinuation-equals-failure, HIV = human immunodeficiency virus,
TE = treatment-experienced, TN = treatment-naïve.
