# Supplementary material for: Twelve-month effectiveness and safety of bictegravir/emtricitabine/tenofovir alafenamide in people with HIV from the Canadian cohort of the observational BICSTaR study
Source: Medicine (Baltimore). 2024 Apr 19;103(16):e37785. doi: 10.1097/MD.0000000000037785 (PMC11029942; doi:10.1097/MD.0000000000037785)
Supplement: Supplementary file 4 [file medi-103-e37785-s004.docx]

**Supplementary Digital Content** **Figure 2.** Changes in lipid levels from baseline to Month 12 in (A) TN participants and (B) TE participants.


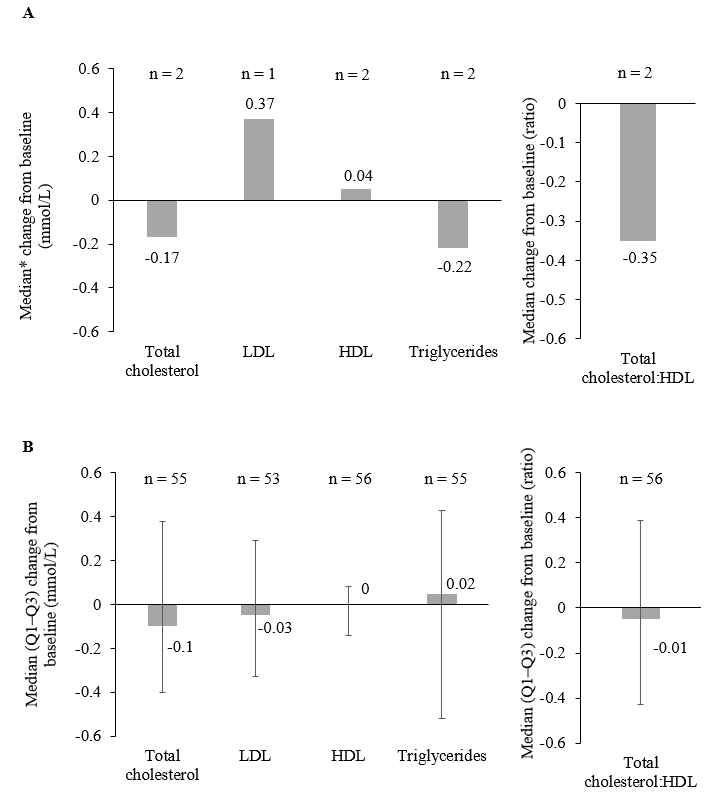


*Q1–Q3 data not shown due to the low participant numbers.

HDL = high-density lipoprotein, LDL = low-density lipoprotein, TE = treatment-experienced, TN = treatment-naïve.
