# Supplementary material for: Twelve-month effectiveness and safety of bictegravir/emtricitabine/tenofovir alafenamide in people with HIV from the Canadian cohort of the observational BICSTaR study
Source: Medicine (Baltimore). 2024 Apr 19;103(16):e37785. doi: 10.1097/MD.0000000000037785 (PMC11029942; doi:10.1097/MD.0000000000037785)
Supplement: Supplementary file 5 [file medi-103-e37785-s005.docx]

**Supplementary Digital Content** **Figure 3.** Changes in eGFR levels from baseline to Month 12 in TN and TE participants.


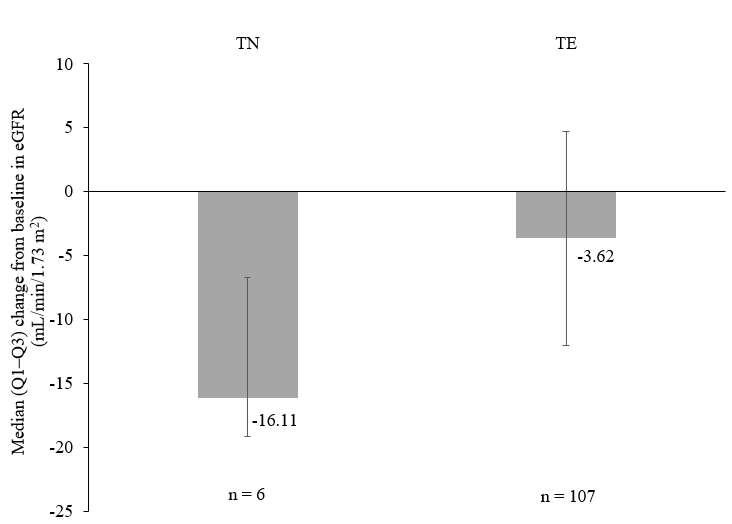


eGFR = estimated glomerular filtration rate, TE = treatment-experienced,
TN = treatment-naïve.
